# Knowledge Reasoning- and Progressive Distillation-Integrated Detection of Electrical Construction Violations

**DOI:** 10.3390/s24248216

**Published:** 2024-12-23

**Authors:** Bin Ma, Gang Liang, Yufei Rao, Wei Guo, Wenjie Zheng, Qianming Wang

**Affiliations:** 1 State Grid Henan Electric Power Company Electric Power Science Research Institute, Zhengzhou 450052, China; binma1995@163.com (B.M.); lganggrid@163.com (G.L.); feiyurao84@163.com (Y.R.); 2 State Grid Henan Electric Power Company, Zhengzhou 450052, China; gaogrid@163.com; 3 Department of Automation, North China Electric Power University, Baoding 071003, China; zhengwj0726@163.com

**Keywords:** electric power construction, violation detection, knowledge reasoning, multi-level distillation, progressive distillation

## Abstract

To address the difficulty in detecting workers’ violation behaviors in electric power construction scenarios, this paper proposes an innovative method that integrates knowledge reasoning and progressive multi-level distillation techniques. First, standards, norms, and guidelines in the field of electric power construction are collected to build a comprehensive knowledge graph, aiming to provide accurate knowledge representation and normative analysis. Then, the knowledge graph is combined with the object-detection model in the form of triplets, where detected objects and their interactions are represented as subject–predicate–object relationship. These triplets are embedded into the model using an adaptive connection network, which dynamically weights the relevance of external knowledge to enhance detection accuracy. Furthermore, to enhance the model’s performance, the paper designs a progressive multi-level distillation strategy. On one hand, knowledge transfer is conducted at the object level, region level, and global level, significantly reducing the loss of contextual information during distillation. On the other hand, two teacher models of different scales are introduced, employing a two-stage distillation strategy where the advanced teacher guides the primary teacher in the first stage, and the primary teacher subsequently distills this knowledge to the student model in the second stage, effectively bridging the scale differences between the teacher and student models. Experimental results demonstrate that under the proposed method, the model size is reduced from 14.5 MB to 3.8 MB, and the floating-point operations (FLOPs) are reduced from 15.8 GFLOPs to 5.9 GFLOPs. Despite these optimizations, the AP50 reaches 92.4%, showing a 1.8% improvement compared to the original model. These results highlight the method’s effectiveness in accurately detecting workers’ violation behaviors, providing a quantitative basis for its superiority and offering a novel approach for safety management and monitoring at construction sites.

## 1. Introduction

Power construction plays a critical role in substations and power transmission and distribution systems, ensuring the safe and reliable operation of the grid [1,2]. However, safety accidents caused by unsafe behaviors or violations during power construction have become a pressing issue in the field [3,4]. In such scenarios, the work environment is often characterized by complexities, such as harsh outdoor conditions, high altitudes, and the need for coordination among multiple teams, which further elevate the risks. Moreover, the diversity in safety equipment—including helmets, safety belts, work clothes, gloves, and other personal protective equipment (PPE) and the variability in worker behaviors add to the challenges of ensuring safety compliance. In particular, in complex power construction environments, if workers neglect to wear personal protective equipment (PPE), such as helmets, safety belts, work clothes, and gloves, it could result in severe safety hazards, even posing a threat to their personal safety [5,6]. Therefore, adhering to safety regulations and correctly using PPE is of paramount importance for safeguarding the lives of power workers [7]. Despite the establishment of safety regulations, the awareness of safety among workers remains insufficient, posing significant challenges to power construction safety. In power construction projects, effectively monitoring and detecting workers’ operational behaviors on-site, and promptly warning of non-compliant actions, are key measures to ensure their safety. In recent years, video surveillance has been widely applied in power construction safety management systems [8]. However, traditional manual detection methods have drawbacks such as high omission rates, low efficiency, labor intensiveness, and low reliability [9]. To address challenges like slow detection of safety behaviors and delayed warnings of violations, real-time detection of compliant equipment usage is essential to prevent accidents. With the increasing application of deep learning-based object-detection algorithms, these methods provide a more reliable and effective way to ensure the safe operation of the power grid [10].

However, despite their advancements, certain challenges remain unaddressed, particularly in adapting these algorithms to the unique demands of power construction scenarios. Firstly, these algorithms often rely on powerful cloud platforms or high-performance GPU clusters for efficient detection, which is not ideal for edge devices, as they generally have limited computing resources and may not handle such computational demands in real time [11]. Secondly, current methods have not fully leveraged domain-specific knowledge in power environments [12], instead using generic object-detection algorithms. Power environments have their own particularities, and if domain knowledge is fully utilized, detection accuracy could be significantly improved. To further enhance the accuracy and applicability of violation detection, lightweight algorithms need to be developed while fully leveraging domain-specific knowledge to achieve more efficient and accurate detection solutions. Such methods would play a more significant role in violation detection in power construction, improving safety and reliability.

To address the issue of detecting violations in power construction environments, this paper proposes an innovative method that integrates knowledge reasoning with progressive multi-level distillation. Firstly, this method specifically targets the characteristics of power construction environments by converting domain-specific knowledge into triplet form, constructing an external prior-knowledge module specifically designed for power construction. This module guides the deep learning network to more effectively detect specific violations in power construction scenes. Secondly, to meet the demands of lightweight and efficient models in power construction environments, this paper employs progressive multi-level distillation to compress and optimize the deep learning network. By distilling at the object level, region level, and global level, this method significantly reduces the loss of contextual information during distillation, while progressive distillation alleviates the gap between the teacher and student models. This approach achieves model lightweighting while ensuring detection accuracy in power construction scenarios.

In summary, the contributions of this paper are as follows:

1. We introduced a knowledge reasoning module based on an electric power-construction knowledge graph, allowing for standardized analysis and accurate representation of compliance behaviors in construction scenarios.
2. We developed a progressive multi-level distillation strategy, which transfers knowledge at multiple levels (object, region, and global), effectively minimizing contextual information loss and enhancing detection accuracy.
3. We achieved a lightweight model design suitable for real-time violation detection in resource-limited construction environments, improving both efficiency and reliability in safety monitoring.

The structure of this paper is as follows: Section 2 presents related work on detection of violations and knowledge graph-based object detection, highlighting the issues present in existing methods. Section 3 provides a detailed introduction to the YOLOv5-based baseline model and its improvements for violation detection in electric power construction scenarios, including the design of the knowledge reasoning module and the progressive multi-level distillation module. Section 4 describes the experimental environment and dataset, along with the evaluation metrics for the model. Section 5 presents the experimental results, comparing the performance of the proposed method with current advanced detection algorithms, thereby validating the advantages of the proposed approach in terms of accuracy and lightweight design. Finally, Section 6 summarizes the research conclusions and discusses potential directions for future improvements.

## 2. Related Work

### 2.1. Detection of Violations

Yang et al. [13] proposed an object-detection model for detecting PPE compliance in power environments, which improved detection performance by embedding an asymmetric convolution module and adding a global attention mechanism in the feature extraction network. Yang et al. [14] introduced a spatiotemporal information fusion network for detecting violations in power production environments, enhancing accuracy through a spatiotemporal attention mechanism in an auxiliary channel. He et al. [15] proposed a violation detection algorithm based on key-point detection and attention mechanisms, improving the accuracy of PPE violation detection by designing a single-person key-point detection model and attention mechanism. Li et al. [16] designed a system for detecting violations at power infrastructure sites, which incorporated the concept of time-shift and attention mechanisms to extract spatiotemporal features from violation data, enhancing the expression of critical detail features.

Despite the advancements achieved by these models, they still exhibit notable limitations, particularly when dealing with complex scenarios and the need for lightweight deployment. While attention mechanisms and spatiotemporal features enhance accuracy, they also increase model complexity and computational overhead, further exacerbating the issue.

### 2.2. Knowledge Graph-Based Object Detection

Jiang et al. [17] proposed Hybrid Knowledge Routed Modules, which represent co-occurrence relationships and spatial position relationships as graph structures to enhance feature representation and improve the reasoning capability of the model. Chen et al. [18] introduced a graph-based relation-aware network that improves feature representation by mining global semantic relationships in labels and local spatial relationships in images, thereby enhancing detection performance. Zhai et al. [19] proposed a cascade-reasoning graph network that utilizes supervised graph learning, graph attention networks, and graph convolutional networks to reason about co-occurrence, semantic, and spatial knowledge, improving model detection performance. Xu et al. [20] developed an adaptive global reasoning model, which generates a global semantic pool by collecting the weights of the previous classification layer for each class and then adaptively enhances each object feature by engaging with different semantic contexts in the global semantic pool.

These methods effectively combine knowledge graphs with object-detection models, improving detection performance. However, they are all implemented on R-CNN series models, and the feature enhancement techniques employed tend to increase the model’s parameter count and computational complexity. This presents challenges for deploying these models on edge devices, where computational resources are typically limited.

## 3. Method

The baseline model used in this paper is YOLOv5, which excels in detection accuracy and is highly suited for deployment in industrial applications due to its robust performance and compatibility with edge devices. YOLOv5 achieves high detection accuracy through multi-scale training and testing strategies, as well as finer feature representations. Additionally, YOLOv5 features efficient training and a compact model, with a simple network structure that maintains accuracy while reducing storage and computational resource overhead, making it suitable for operation on resource-constrained devices. Its modular design is easy to understand, extend, and optimize to adapt to different application scenarios.

**Figure 1:**
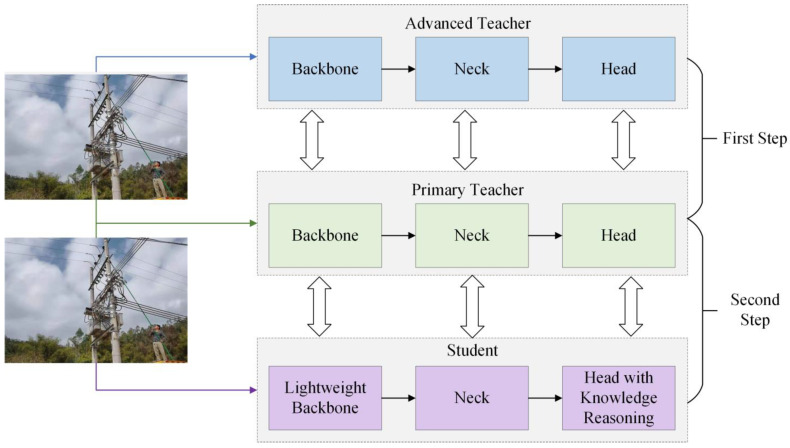
Model structure.

The categories detected in this paper include the following: Safety Harness Fastened, Safety Harness Not Fastened, Using Phone, Not Using a Safety Harness, Wearing Safety Helmet, and Not Wearing Safety Helmet. These behaviors are commonly observed in power construction scenarios, where safety precautions are crucial. By focusing on these specific actions, the model aims to identify key safety-related behaviors that are critical for ensuring worker safety in high-risk environments. To further improve the detection of violations and achieve model lightweighting, this paper builds upon YOLOv5 by integrating knowledge reasoning and progressive multi-level distillation techniques. The model structure is shown in Figure 1.

The feature extraction network is used to extract the foundational feature representations of power construction scenes and personnel violations from the images. In the neck network, these initial features are further integrated and optimized, while in the prediction network, the model performs the object-detection tasks. We have made improvements to the YOLOv5 model to meet the needs of detecting personnel violations.

First, a lightweight feature extraction network is introduced to reduce the number of model parameters while ensuring effective extraction of key visual features in power construction scenes. Second, the relationships between objects in the power construction scene are expressed in the form of triplets, enabling adaptive global reasoning for specific relationships or similar attribute categories. This enhances the model’s ability to detect violations in complex construction environments.

Finally, a highly accurate but complex model is used as the teacher model, which is further divided into advanced and primary teacher models based on complexity. The lightweight model proposed in this paper is used as the student model. Through multi-level progressive knowledge distillation, the student model learns the knowledge from the teacher models more effectively, thereby improving the model’s detection performance.

The following sections will introduce the knowledge reasoning module and the multi-level progressive distillation module in detail.

### 3.1. Knowledge Reasoning Module

As the data advantage of deep learning diminishes, integrating external knowledge has become an important direction for improving model performance. In the field of computer vision, external knowledge is often embedded in forms such as object attributes, labeled relationships, spatial positions, and co-occurrence relationships. Some studies use relational and shared attribute knowledge for large-scale object detection [21,22], while others use semantic-space attribute similarities to guide target training. Typically, these external knowledge sources require large amounts of data for support.

In this paper, we construct an external knowledge module specific to the power construction field, inspired by WordNet. Regulations and standards are organized into a corpus, and a tree structure is used to organize different types of vocabulary. First, extensive literature review and data collection were conducted to cover regulations, standard operating procedures, and safety guidelines in the power construction domain. After text cleaning, classification, and annotation, we built a knowledge base, and the tree structure was used to visually represent concepts and their relationships, where each node represents a concept or category, and the edges indicate relationships between concepts. To integrate this knowledge base into the YOLO framework, we enhance the model by introducing a knowledge-guided feature extraction module. This module incorporates external knowledge into the feature maps during object detection, effectively enabling the model to focus on semantically relevant features. For example, when detecting safety helmets, the model uses the associated knowledge to suppress irrelevant background information and emphasize key object attributes.

Our knowledge base focuses on integrating power construction safety-related content, including safety equipment usage, high-altitude work standards, and power equipment operation regulations. To ensure its practicality and accuracy, the knowledge base was reviewed by experts. Ultimately, this knowledge base became an integral part of the deep learning model, providing rich domain-specific information and improving the accuracy of violation detection. The knowledge base is constructed in the form of triplets, where each triplet includes two concepts or entities and their relationship. It covers entities such as tools, equipment, and safety gear, along with their attributes and associations, to support the model’s reasoning and analysis.

**Figure 2:**
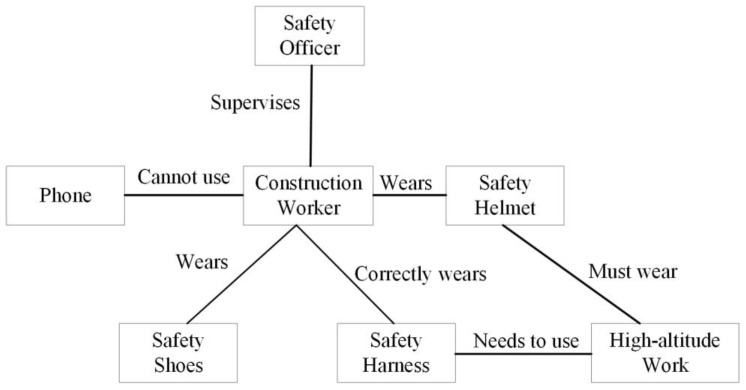
Example of triplet relationships.

As shown in Figure 2, a specific relationship could be “construction worker”–“cannot use”–“phone”; “safety officer”–“supervises”–“construction worker”; “high voltage area”–“requires”–“special safety measures”; “safety shoes”–“used for”–“protective work”; “safety harness”–“used for”–“working at heights”. For example, in the triplet ”construction worker”–“must comply with”–“wear a safety helmet”, ”construction worker” is an entity representing the worker, “wear a safety helmet” is an entity representing a safety rule, and “must comply with” is the relationship between the two. This indicates that one of the safety rules that construction workers must follow in power construction environments is wearing a safety helmet. In the YOLO model, the triplets derived from the knowledge base are used to enhance the object classification layer. Specifically, we augment the object confidence scores by combining them with semantic relevance scores calculated from the knowledge graph. This ensures that objects with stronger semantic associations to the task are given higher detection priorities.

In the power construction scenario, the core of knowledge integration and ontology construction is handling specialized terminology and concepts related to construction safety through semantic similarity and hierarchical relationships. We transformed the categories and relationships found in power construction standards—such as safety rules, operating procedures, working environments, and personal protective equipment—into a graphical data structure. This structure clearly presents the connections between different elements, making it easier for the external knowledge module to utilize.

**Figure 3:**
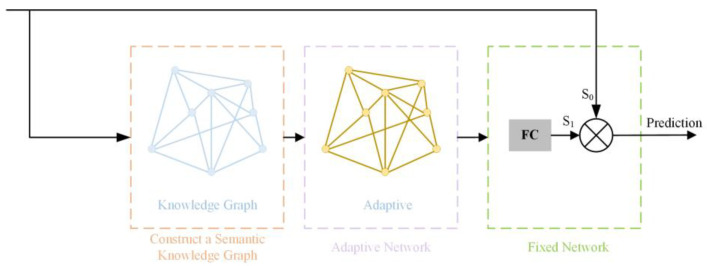
External-knowledge reasoning module.

By constructing the knowledge module in this way, the model can better understand specific terms and concepts related to the power construction field. For example, it can recognize safety equipment (such as helmets and safety goggles) and understand their roles in ensuring safety, as well as grasp the requirements for safety measures in different construction environments. This approach significantly improves the accuracy and stability of the model when detecting violations and enhances its effectiveness in complex construction scenarios. The external knowledge module is illustrated in Figure 3.

The external knowledge constructed in this paper requires the following three steps.

Step 1: Constructing a Semantic Knowledge Graph. The expression of object categories in the knowledge graph is represented as *G = (V,E)*, where *V* denotes the category concept nodes and *E* represents the paths connecting different nodes. The knowledge graph we use is based on regulations and standards in the power construction field, covering concepts such as safety measures, tool usage, operational procedures, and work area safety standards, along with their complex relationships. This structured information can be effectively mapped to object categories, aiding in the detection of safety violations and the understanding of tool usage regulations.

**Figure 4:**
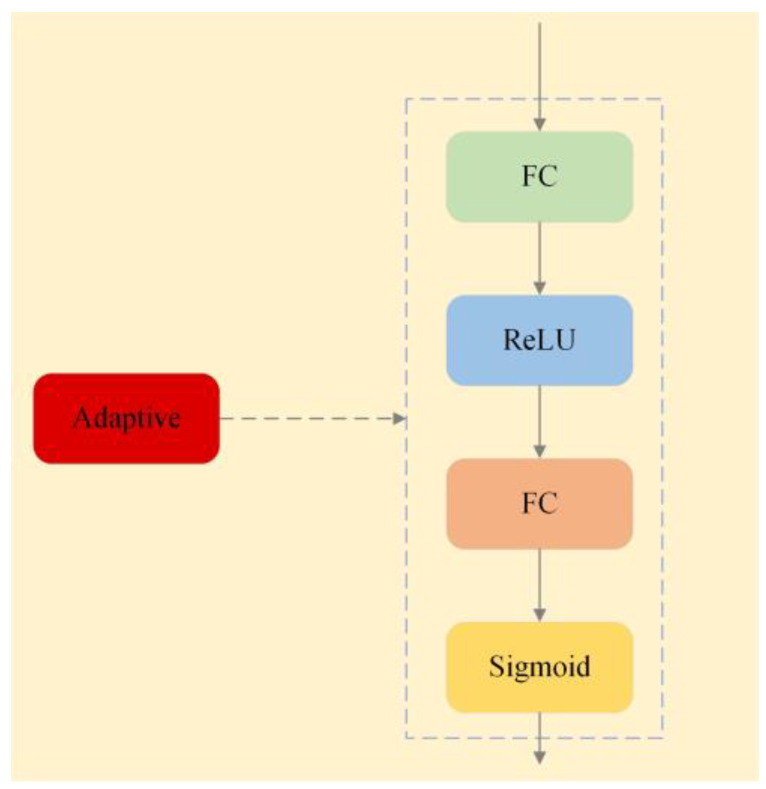
Adaptive connection network.

Step 2: Establishing an Adaptive Connection Network. This network addresses the issue of redundancy and noise that may arise from directly embedding the semantic knowledge graph, particularly in the dynamic and complex environment of power construction. The network adaptively activates the knowledge graph and dynamically adjusts the portions of the knowledge used according to each scene. For example, in high-altitude work scenarios, the network tends to activate knowledge related to high-altitude safety measures, while for ground-level operations, it focuses on ground safety regulations. The adaptive connection network is shown in Figure 4.

The adaptive connection network is similar to the gating mechanism in recurrent neural networks. This mechanism allows the network to adapt more flexibly to changes in various scenarios, such as different work environments (e.g., high-altitude operations, ground-level work), the use of safety equipment, and complex human interactions. Conversely, for ground-level cable-laying operations, it prioritizes features like ground markers and protective railings.

Additionally, the network explicitly models the correlation between feature channels by learning parameters. The formula for adaptive connection is shown as follows:

(1) $$\varepsilon =F_{adptive}(R,W_{a})=\delta (W_{2}\delta (W_{1}R))$$

In Formula (1), the parameters are defined as follows: *R* is the input feature vector provided to the network. *W_1_* is a learnable weight matrix with dimensions C/r*C, where *C* is the number of input channels, and *r* is a scaling parameter introduced to reduce the number of channels, thereby decreasing the computational complexity. After the transformation by *W_1_*, the data are passed through a ReLU activation function δ to introduce non-linearity. Next, *W_2_*, a weight matrix with dimensions C*C/r is applied to restore the original dimensions of the feature map. Finally, a Sigmoid function is applied to produce the final output, which provides the adaptive weights for each feature channel. Together, these parameters enable the network to efficiently learn and model features of channel correlations while minimizing computational costs.

Step 3: Revising the Semantic Knowledge Graph. The adaptive knowledge graph R is processed through a fully connected layer to obtain S, which is then multiplied by the original features for weighted adjustment. When *S_1_* > 1, it indicates that the current features are highly correlated with the important semantic features in the power construction scene. As a result, the network will adaptively enhance these semantic features, such as emphasizing critical information like safety helmets, safety harnesses, and high-voltage area signs. This helps the model more accurately detect and understand key elements and potential safety risks in power construction environments.

When *S_1_* < 1, it signifies that the current features are less related to the main semantic features of the power construction scene. The network will automatically suppress these less relevant features and filter out possible noise. This prevents the model from over-focusing on non-essential or distracting information in the power construction scene, such as background noise or irrelevant objects. The calculation formula is as follows:

(2) $$S=S_{1}•S_{0}=\varepsilon W_{e}•S_{0}$$

### 3.2. Progressive Multi-Level Distillation

The entire knowledge distillation network consists of two teacher networks and one student network. YOLOv5m and YOLOv5s are used as the advanced teacher network and the primary teacher network, respectively. The backbone of YOLOv5s is replaced with the lightweight Mobilenetv2 [23], serving as the student network. However, while reducing parameters, the lightweight student network may experience a decline in the accuracy of detecting personnel violations. To mitigate the impact of model compression on accuracy, we employ progressive multi-level knowledge distillation techniques.

Progressive distillation improves the student model’s learning outcomes through a step-by-step process involving two stages of distillation.

In the first stage, the pre-trained advanced teacher model is frozen, and the primary teacher model undergoes training. The primary teacher learns from the advanced teacher’s representational capacity to better guide the student model.

In the second stage, the primary teacher is used to teach the student model. At this point, the primary teacher’s parameters are frozen, and only the student network’s parameters are updated through backpropagation.

Multi-level distillation occurs at the object level, region level, and global level, significantly reducing the loss of contextual information during distillation. Through this approach, the student network effectively learns the knowledge of the teacher networks, including the ability to understand and handle complex situations in power construction scenarios.

**Figure 5:**
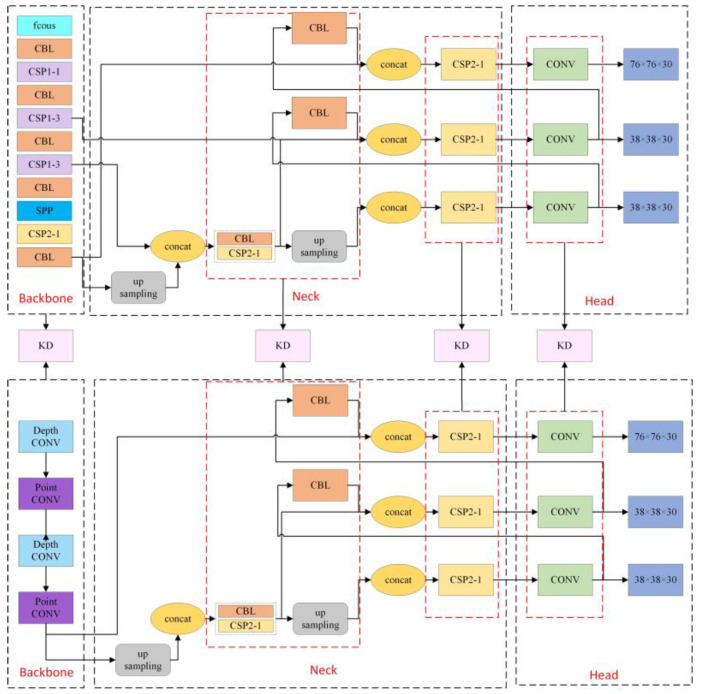
Multi-level knowledge-distillation network structure.

This multi-level knowledge distillation structure not only enhances the student network’s accuracy in detecting violations in power construction environments but also maintains the model’s lightweight characteristics. Figure 5 illustrates the multi-level knowledge distillation structure, using the primary teacher network and student network as examples.

In power construction scenarios, we designed a knowledge transfer method that addresses the complexity and diversity of the environment. This method takes into full consideration the varying conditions and details of the construction environment, such as safety equipment (helmets, safety harnesses), work environments (high-altitude operations, underground cable laying), and safety behavior regulations (correct use of tools, compliance with procedures). Before knowledge distillation, we connect and analyze the feature streams from multiple teacher networks, calculate their importance, and assign different weights.

Let the ith subnetwork of the teacher and student streams be denoted as *i*(*t*) and *i*(*s*), respectively, where *i*(*t*) extracts discriminative features from construction data, progressively moving from basic safety equipment detection to more complex safety-behavior pattern detection. In the backbone stage, the teacher network extracts features after Focus and CBL modules, and through CSP1_X, it performs downsampling to obtain multi-scale features (608 × 608, 304 × 304, 152 × 152, etc.). The neck network performs upsampling and fusion through the FPN and PAN structures, enhancing the detection of small objects and details. The features learned by the teacher network are used to guide the training of the student network, optimizing feature representation and transmission, which further improves detection performance at the prediction stage.

During the distillation process, we apply the SKNet structure, cascading different feature maps to obtain a fused-feature map. This cascading fusion process takes into account the diversity and complementarity of the feature maps, allowing the final fused-feature map to comprehensively represent the critical visual information in power construction scenarios. For example, smaller-scale feature maps may focus more on capturing detailed information, such as hand movements or the use of specific safety equipment by workers, while larger-scale feature maps might be better suited for capturing the overall layout of the scene and distant objects. Subsequently, the fused-feature map passes through average pooling and fully connected layers to generate the vector Z.

By initializing matrices A and B, we generate the corresponding weight matrices a and b, which provide the importance coefficients for the information. The calculation formula is as follows:

(3) $$a=\frac{e^{AZ}}{e^{AZ}+e^{BZ}}$$

(4) $$b=\frac{e^{BZ}}{e^{BZ}+e^{AZ}}$$

Let i(s) represent the features extracted by the student network, which are matched with the output of i(t) from the teacher network. From the description above, we understand that distillation of a single output feature lacks constraints on the intermediate layers. In other words, the features judged by the teacher network have not been fully learned by the student network, which can easily lead to overfitting. To address this, we extract the output-feature knowledge, relational-feature knowledge, and intermediate-feature knowledge from different stages of the teacher network.

These relational and intermediate features are then fused with the corresponding i(s) through four different scales of average pooling layers. This process allows us to calculate the loss function in the network structure, denoted as TtL1−TtL4. The loss function is defined as follows:

(5) $$\begin{array}{l} {\mathrm{TtL}}_{i}{=L}_{2}{(\mathrm{ap}}_{1}{\;(\mathrm{FM}}_{t}{,\;\mathrm{FM}}_{s})\;\\ {+L}_{2}{\;(\mathrm{ap}}_{2}{\;(\mathrm{FM}}_{t}{,\;\mathrm{FM}}_{s}))\;\\ {+L}_{2}{\;(\mathrm{ap}}_{3}{\;(\mathrm{FM}}_{t}{,\;\mathrm{FM}}_{s}))\;\\ {+L}_{2}{\;(\mathrm{ap}}_{4}{\;(\mathrm{FM}}_{t}{,\;\mathrm{FM}}_{s})) \end{array}$$

where L_2_ represents the L_2_ loss function, which is used to measure the difference between the teacher and student network outputs, FM_t_ contains feature-map data from different network layers of YOLOv5s, which are extracted by the teacher network, and FM_s_ refers to the features extracted by the student network.

## 4. Experimental Design

### 4.1. Experimental Environment and Dataset

For the model parameters, we set the batch size to 16, the learning rate to 0.01, and the number of epochs to 200. The experimental environment includes training and testing conducted on an NVIDIA RTX 3090 GPU, using Ubuntu 18.04.1 LTS as the operating system. CUDA 11.2 was used to accelerate the training process, the programming language was Python 3.6, and the network framework was PyTorch.

This study focuses on detecting safety behaviors in power construction environments, a task crucial to ensuring safety at construction sites. Our objective is to use efficient artificial intelligence algorithms to detect key safety behaviors, such as wearing a safety helmet and ensuring that workers’ safety harnesses are properly fastened. To achieve this goal, we constructed a comprehensive and practical dataset using images captured by surveillance cameras at various power construction sites.

During the dataset construction process, we carefully selected and annotated a wide range of images to ensure the accuracy and diversity of the dataset. The final dataset contains six categories: wearing a safety helmet, not wearing a safety helmet, not using a safety harness, safety harness fastened, safety harness not fastened, and using a phone. These categories cover the most common key safety behaviors in power construction scenarios, which are essential for maintaining site safety.

**Figure 6:**
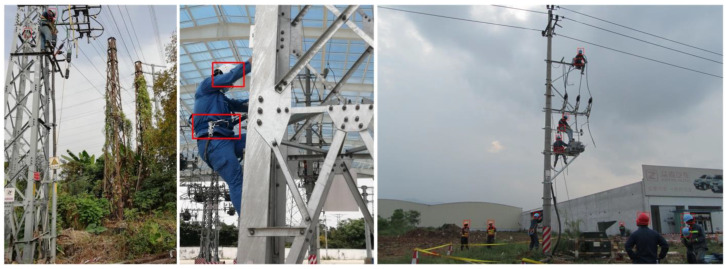
Samples from the violation detection dataset. (The red rectangular box indicates the target).

The dataset consists of 4000 raw images, with the following label counts: 2230 for “Safety Harness Fastened”, 1965 for “Safety Harness Not Fastened”, 1890 for “Using Phone”, 1665 for “Not Using a Safety Harness”, 3874 for “Wearing Safety Helmet”, and 2689 for “Not Wearing Safety Helmet”. Examples from the dataset are shown in Figure 6.

### 4.2. Evaluation Metrics

To quantitatively demonstrate the effectiveness of the algorithm developed in this paper, we use common metrics in current object-detection models: Precision (P), Recall (R), and Average Precision (AP). The AP evaluation metrics include AP50:95 and AP50. The formulas for calculating these metrics are as follows:

(6) $$P=\frac{\mathrm{TP}}{\mathrm{TP}+\mathrm{FP}}$$

(7) $$R=\frac{\mathrm{TP}}{\mathrm{TP}+\mathrm{FN}}$$

(8) $$AP=\int_{0}^{1}p(r)dr$$

where TP (True Positive) represents the number of correctly detected and classified positive examples, FP (False Positive) represents the number of background examples incorrectly detected as objects, and FN (False Negative) represents the number of positive examples that were either classified incorrectly or not detected.

## 5. Experimental Results and Analysis

### 5.1. Comparison of Different Methods

We compared several state-of-the-art object-detection algorithms to evaluate the performance of the proposed method. The methods used for comparison include SSD [24], YOLOv3 [25], YOLOv5, YOLOv7 [26], YOLOv8, and Faster R-CNN [27]. The detection results are shown in Table 1, which illustrates the effectiveness of these algorithms in detecting violations by construction workers in power construction scenarios.

Particular attention was given to each algorithm’s performance in terms of detection accuracy, processing speed, and the ability to detect small objects. This comparison provides a clear understanding of how different models handle the complexities of detecting safety violations in power construction environments.

The data in Table 1 clearly demonstrate the excellent performance of the proposed model in the task of detecting worker safety behaviors in power construction scenarios. Compared with the baseline model YOLOv5, our model achieves a 1.0% improvement in precision, a 0.9% increase in recall, a 3.9% increase in AP50:95, and a 1.8% increase in AP50. These improvements are crucial for safety monitoring in power construction, as even small gains in accuracy can significantly reduce safety risks in high-risk construction environments.

What is more remarkable is that while improving detection performance, our model also achieved significant lightweight optimization. The model size was reduced from 14.5 MB to 3.8 MB, and the floating point operations (FLOPs) decreased from 15.8 to 5.9. This lightweight model, when deployed on resource-constrained devices (such as portable or edge computing devices commonly used on power construction sites), not only provides efficient computational performance but also conserves energy. This is critical for real-time monitoring and long-term operation in power construction environments.

To further compare the effectiveness of different models in detecting worker safety behaviors, we evaluated the detection accuracy (AP50) across six categories: wearing a safety helmet, not wearing a safety helmet, not using a safety harness, safety harness fastened, safety harness not fastened, and using a phone. The detection results for each category are presented in Table 2.

**Figure 7:**
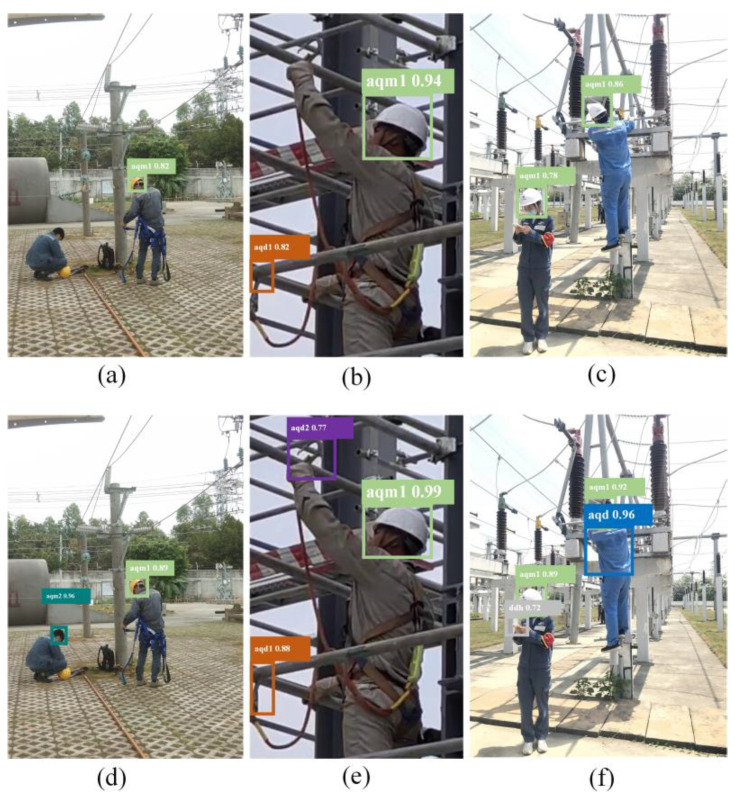
Visualization results of different model detections. (**a**–c) are YOLOv5, (**d**–**f**) are the method proposed in this paper.

As shown in Table 2, the proposed method achieves the best detection performance across all categories. Specifically, for the four violation behaviors—safety harness not fastened, using a phone, not using a safety harness, and not wearing a safety helmet—the AP50 improves by 5.1%, 0.7%, 0.3%, and 3.3%, respectively, compared to the baseline model. The visualized detection results of the model are displayed in Figure 7, where (a)–(c) show the detection results of YOLOv5, and (d),(e) show the detection results of the proposed method.

### 5.2. Ablation Study

The improvements in this paper primarily include the lightweight-feature extraction network, the knowledge reasoning module, and the knowledge distillation module. To further verify the actual contribution of these three methods to the model, we conducted ablation experiments. In these experiments, we added each module step by step and observed its impact on the model’s performance. The results of the ablation study are shown in Table 3.

As shown in Table 3, the three methods—lightweight-feature extraction network, knowledge reasoning module, and multi-level knowledge distillation—all contribute positively to both detection accuracy and model lightweighting.

The lightweight-feature extraction network significantly reduces the model’s floating-point operations (FLOPs) and model size, making it easier to deploy the model on edge devices. However, the lightweight network struggles to fully extract the object features, which negatively affects the model’s accuracy.

The introduction of the knowledge reasoning module helps the model learn structured knowledge specific to power construction scenes, thereby improving its ability to detect worker safety behaviors. However, this module increases the model’s complexity, to some extent.

The multi-level knowledge distillation enables the teacher network to more effectively guide the student network in learning distilled key knowledge and reduces information loss during knowledge transfer. This enhances model performance without adding complexity to the model.

Therefore, the combined application of these three methods not only improves detection accuracy, but also achieves model lightweighting.

To further validate the effectiveness of the distillation losses in the multi-level knowledge distillation, we designed an ablation experiment with four individual distillation-loss functions. Each distillation loss was applied separately, and the resulting detection performance is shown in Table 4. This experiment highlights the contribution of each loss function in guiding the student network to learn from the teacher network and improving detection accuracy.

As shown in Table 4, each distillation loss improves the model’s detection performance by transferring the knowledge learned by the teacher network to the student network. Applying multi-level distillation minimizes the loss during the knowledge transfer process, achieving the best learning outcomes. This approach distills knowledge at different levels, reducing potential information loss in the transfer process to the maximum extent.

For example, at lower levels, the model might focus on learning basic visual features and patterns, such as the shape and color of safety helmets and harnesses. At higher levels, it can capture more complex behavior patterns, such as unsafe work postures or potential risky behaviors.

**Figure 8:**
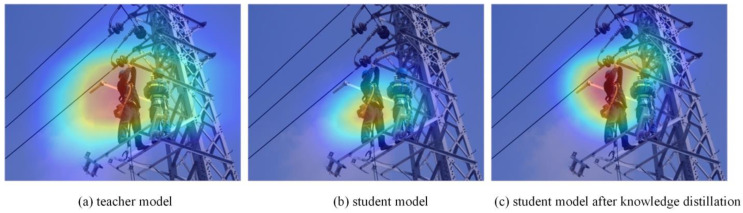
Feature map visualization.

To better demonstrate the effectiveness of multi-level knowledge distillation, we visualized the feature maps of the teacher model, the original student model, and the student model after knowledge distillation, as shown in Figure 8.

It is evident that after applying knowledge distillation, the model becomes more focused on the target and its corresponding regions. This highlights how the distillation process enhances the model’s ability to capture relevant features, improving its overall detection performance.

To verify the effectiveness of progressive distillation, we conducted experiments using different teacher models. The results are shown in Table 5, and further demonstrate the impact of selecting various teacher models on the performance of the student network. This comparison highlights how different teacher models contribute to guiding the student network in learning more effectively, improving overall detection accuracy.

It can be observed that compared to directly using a single advanced-teacher or primary-teacher model for knowledge distillation, the progressive distillation strategy achieves better results. Through phased learning, progressive distillation first leverages the rich expressive capabilities of the advanced teacher model to establish a solid knowledge foundation for the primary-teacher model. Subsequently, the primary teacher gradually guides the student model, enabling effective knowledge transfer.

This multi-stage process ensures that the student model can effectively absorb knowledge from both teacher models, leading to improved performance in complex scenarios such as detecting safety behaviors in power construction environments. The gradual knowledge transfer helps mitigate the gap between the advanced and student models, optimizing both the learning efficiency and detection accuracy.

### 5.3. Public Dataset Experiments

To further validate the generalizability and adaptability of the proposed knowledge distillation method, experiments were conducted on the RSOD public dataset, and the results are shown in Table 6. The RSOD dataset includes four categories: airplane, playground, overpass, and oil tank.

As shown in Table 6, the knowledge distillation strategy proposed in this paper is more effective in facilitating knowledge transfer, which in turn improves the model’s detection performance. This demonstrates that the enhanced distillation approach leads to better learning and understanding of the task, resulting in higher accuracy and more reliable detection results.

## 6. Conclusions

The experimental results indicate that the proposed solution based on knowledge reasoning and progressive multi-level distillation is highly effective in detecting violations by construction workers in power construction scenarios. By integrating the power construction knowledge graph with deep learning networks, the detection accuracy and speed were significantly improved. In particular, the network optimized by the progressive multi-level distillation technique allows the model to perform efficient and accurate detection on edge devices, which is crucial for real-time monitoring and safety management at construction sites. We also conducted experiments on the edge device Jetson Xavier NX, and the results show that our lightweight model is capable of detecting 30 images per second, making it suitable for real-time applications. However, there is still room for improvement. First, the deep learning network structure could be further optimized to enhance detection capability in more complex scenarios. Second, enriching the content of the knowledge graph with more regulations and domain-specific knowledge could improve the model’s precision and robustness. Additionally, exploring other model compression and optimization techniques will be key to increasing efficiency in edge devices. Future research will focus on refining and extending this approach, validating its effectiveness in detecting violations in other fields.

Future work will focus on refining these aspects, particularly by optimizing the deep learning network for more challenging environments and expanding the knowledge graph to cover a broader range of regulations. Additionally, we aim to investigate alternative optimization techniques and validate the proposed method in other domains, such as industrial safety or urban construction monitoring.

## Figures and Tables

**Table 1:** Personnel safety behavior detection results.

Method	P	R	AP50:95	AP50	Model Size/MB	GFLOPS
SSD	84.6	80.3	48.7	82.5	182.0	30.5
YOLOv3	89.6	88.2	56.1	88.6	470.2	154.9
YOLOv5	92.4	89.2	56.3	90.6	14.5	15.8
YOLOv7	91.3	89.8	54.6	89.8	103.2	71.3
YOLOv8	90.6	89.6	57.3	90.2	16.5	28.8
Faster R-CNN	90.2	87.1	52.8	87.2	528	63.9
Ours	93.4	90.1	60.2	92.4	3.8	5.9

**Table 2:** Detection results for different categories.

Method	Safety Harness Fastened	Safety Harness Not Fastened	Using Phone	Not Using a Safety Harness	Wearing Safety Helmet	Not Wearing Safety Helmet
SSD	83.4	80.2	86.8	78.6	90.2	75.8
YOLOv3	86.7	89.2	85.3	90.1	88.7	91.6
YOLOv5	90.8	90.6	89.6	91.3	92.4	88.9
YOLOv7	89.7	89.6	90.4	90.8	88.2	90.1
YOLOv8	89.1	88.6	89.4	90.5	92.4	91.2
Faster R-CNN	82.8	85.2	86.4	88.3	90.2	90.3
Ours	91.2	95.7	90.3	91.6	93.4	92.2

**Table 3:** Ablation experiment for the three modules. (The “√” symbol indicates that the method is used).

Lightweight-Feature Extraction Network	Knowledge Reasoning	Knowledge Distillation	AP50	Size	GFLOPS
			90.6	14.5	15.8
√			88.9	2.8	3.6
	√		94.2	16.2	17.4
√		√	91.4	14.5	15.8
√	√		90.8	3.8	5.9
√	√	√	92.4	3.8	5.9

**Table 4:** Ablation experiment for distillation losses. (The “√” symbol indicates that the method is used).

KD1	KD2	KD3	KD4	AP50
				88.9
√				89.2
	√			89.7
		√		89.4
			√	89.5
√	√	√	√	90.6

**Table 5:** Ablation experiment for progressive distillation.

Teacher	Student	AP50
YOLOv5s	/	90.6
YOLOv5m	/	92.8
/	YOLOv5s-Mobilenetv2	88.9
YOLOv5s	YOLOv5s-Mobilenetv2	90.2
YOLOv5m	YOLOv5s-Mobilenetv2	90.4
YOLOv5-m→s	YOLOv5s-Mobilenetv2	91.2

**Table 6:** RSOD dataset experiments.

Teacher	Student	AP50
YOLOv5s	/	95.5
YOLOv5m	/	96.8
/	YOLOv5s-Mobilenetv2	92.6
YOLOv5s	YOLOv5s-Mobilenetv2	93.2
YOLOv5m	YOLOv5s-Mobilenetv2	93.8
YOLOv5-m→s	YOLOv5s-Mobilenetv2	94.4
YOLOv5-m→s + KD1–4	YOLOv5s-Mobilenetv2	94.7

## Data Availability

We are sorry that the power data cannot be disclosed, due to their particularity and confidentiality; further inquiries can be directed to the corresponding authors.
